# Features of Galvanostatic Electrodeposition of NiFe Films with Composition Gradient: Influence of Substrate Characteristics

**DOI:** 10.3390/nano12172926

**Published:** 2022-08-25

**Authors:** Tatiana I. Zubar, Tatsiana I. Usovich, Daria I. Tishkevich, Oleg D. Kanafyev, Vladimir A. Fedkin, Anna N. Kotelnikova, Maria I. Panasyuk, Alexander S. Kurochka, Alexander V. Nuriev, Abubakr M. Idris, Mayeen U. Khandaker, Sergei V. Trukhanov, Valery M. Fedosyuk, Alex V. Trukhanov

**Affiliations:** 1 Laboratory of Magnetic Films Physics, Scientific-Practical Materials Research Centre of National Academy of Sciences of Belarus, 220072 Minsk, Belarus; 2 Laboratory of Single Crystal Growth, South Ural State University, 454080 Chelyabinsk, Russia; 3 Department of Electronic Materials Technology, National University of Science and Technology MISiS, 119049 Moscow, Russia; 4 Department of Chemistry, College of Science, King Khalid University, Abha 62529, Saudi Arabia; 5 Research Center for Advanced Materials Science (RCAMS), King Khalid University, Abha 62529, Saudi Arabia; 6 Centre for Applied Physics and Radiation Technologies, School of Engineering and Technology, Sunway University, Bandar Sunway 47500, Malaysia; 7 Department of General Educational Development, Faculty of Science and Information Technology, Daffodil International University, DIU Rd, Dhaka 1341, Bangladesh

**Keywords:** NiFe films, electrodeposition, chemical composition, microstructure, roughness

## Abstract

NiFe films with a composition gradient are of particular interest from the point of view of fundamental science and practical applications. Such gradient magnetic structures may exhibit unique functional properties useful for sensory applications and beyond. The issue surrounds the anomaly concerning the compositional gradient formed near the substrate in electrolytically deposited binary and ternary iron-containing alloys, which has not previously been clearly explained. In this work, light is shed on this issue, and a clear relationship is found between the structure and surface properties of the substrate, the initially formed NiFe layers and the film composition gradient.

## 1. Introduction

Permalloy, or an alloy based on Ni and Fe (less than 50 wt.%), is one of the most common soft magnetic alloys for practical applications due to the optimal combination of electrical, magnetic and operational properties [1,2,3,4]. The low cortical force, high magnetization and magnetic permeability in combination with almost zero magnetostriction make these materials attractive for use as magnetic-field sensors [5], electromagnetic shields [6,7], inductor cores for electromagnets [8], magnetic recording heads [9,10,11,12], inductor cores [13,14], microwave noise filters [15,16], tunable noise suppressors [17] and much more. The role of NiFe films and coatings in the production of new nanodevices (such as microelectromechanical MEMS, NEMS) is significant [5,18,19].

Electrolytic deposition is widely used in industry for applying various types of metal and composite coatings due to the economic efficiency of the method [20,21,22]. Electrolytically deposited films and coatings based on NiFe alloys are still the most commonly used materials for electromagnetic shields [23,24,25] due to their high magnetic permeability and manufacturability of deposition onto complex structural surfaces. In connection with the above, nickel and iron alloys, or permalloy alloys, have been widely studied for several decades, including the features of the synthesis of films and coatings by electrolytic deposition [26,27,28,29]. Despite the apparent simplicity of the electrolytic deposition method, the relationship between the technological parameters of synthesis and the structure, composition and properties of the resulting coatings is complex and difficult to predict [30,31,32].

It has been observed by many authors that iron exhibits anomalous coprecipitation in binary and ternary alloys during electroplating [33,34,35,36]. The molar fraction of Fe in the deposited film is greater than the corresponding ratio of concentrations in the solution, taking into account the electrode potentials. Another anomalous aspect is the presence of an iron content gradient in binary and ternary alloys [29,34,37,38,39,40,41]. Iron-rich layers near the substrate are formed in the galvanostatic [37,42] and potentiostatic regimes [43] at direct [44] and pulsed current modes [45,46] in electrolytes of various compositions [44,45,47]. In all publications studied by us, after the formation of a certain thickness of the film (hundreds of nanometers or a few micrometers), the composition became constant and did not change during further film growth. The authors gave various explanations for this phenomenon, which became a regularity in the electrolytic deposition of iron-containing films, but they did not reach a conclusion.

Previous attempts have also been made to find a relationship between chemical composition gradient and substrate roughness [35]. However, the influence of surface properties such as wettability, surface energy and the ratio of total to nominal area was not taken into account. Therefore, in this work, we decided to apply an approach not previously used to study the effect of the structure and surface properties of the substrate on the composition gradient of NiFe films. The task of searching for a strict correlation between the synthesis conditions, taking into account the characteristics of the substrate, is extremely important because it will allow controlled synthesis of electrodeposited films with a composition gradient, which can have unique functional properties. The anomalous and extremely promising properties of gradient systems based on NiFe have already been noted in publications [34,39,48,49,50,51,52], but the composition gradient was always random and not planned in advance.

## 2. Materials and Methods

Electrolyte deposition was used to obtain samples of NiFe films. The substrates for the electrodeposition were three types of copper foil with different preparations. The first type was a copper foil mechanically polished using diamond paste with abrasive particle sizes of 3 and 0.25 μm. The remains of the paste were removed with trichlorethylene. The surface was degreased with Viennese lime, and the oxide layer was removed with a 5% solution of hydrochloric acid for 5 s. The second type of substrate was not polished. The surface was etched in HCl (5%) for 5 s. This preparation removes impurities and oxide layers, but traces of rolled copper and different defects remain on the surface. The third type of substrate used was degreased and etched in a harder solution of ammonium persulfate—(NH_4_)_2_SO_8_—for 1 min. Ammonium persulphate quickly removed the top layer of copper foil and smoothed out the rolling marks.

**Figure 1:**
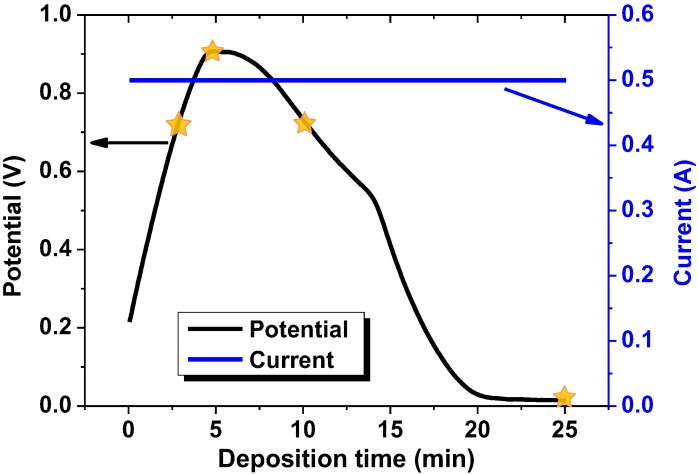
Potential (black line) change during NiFe film synthesis in galvanostatic electrodeposition mode. The current was constant (blue line).

Four NiFe films were deposited onto each type of substrate with a galvanostatic electrodeposition mode. The potential change during NiFe film deposition is demonstrated in Figure 1. The samples differed by deposition time, which was 1, 3, 10 and 25 min (corresponding to the potential values marked with stars in Figure 1). More information about substrate preparation and sample features is given in Table 1. The complex electrolyte for NiFe deposition contained NiSO_4_ 7H_2_O (250 g/L), NiCl_2_ 6H_2_O (20 g/L), H_3_BO_3_ (25 g/L), MgSO_4_ 7H_2_O (110 g/L), FeSO_4_ 7H_2_O (35 g/L), D (+) Glucose (85 g/L), HC_6_H_7_O_6_ (3 g/L) and saccharin (3 g/L) [53,54]. The solution temperature was kept at 35 °C and the pH level at 2.0. The current density was 25 mA/cm^2^. Potentiostat–galvanostat R-45X (Chernogolovka, Russia) working in galvanostatic mode was used for film deposition.

Surface microstructure was investigated using a scanning electron microscope (SEM) Zeiss EVO 10 (Zeiss, Oberkochen, Germany) and atomic force microscope (AFM) NT-206 (Microtestmachines, Gomel, Belarus). SEM images were obtained with a second electron detector at an accelerating voltage of 20 kV. The contact scanning mode was used to obtain AFM images [55,56,57,58,59]. A silicon tip with a curvature radius of 10 nm and a force constant of 0.6 N/m was used during AFM scanning. Chemical composition was studied using energy-dispersive X-ray spectroscopy with AZtecLive Advanced and Ultim Max 40 (Oxford Instruments, Bognor Regis, UK) at an accelerating voltage of 5 kV. The surface roughness (*Ra*) was estimated by using the following equation

(1) $$R_{a}=\frac{\int_{0}^{L}\left|r\left(x\right)\right|dx}{L},\;$$

where *r* (*x*) is the deviation of the profile from its mean and *L* is the sample length. At least three AFM images were used for the calculation of surface roughness and the ratio of the nominal/full area [60]. Specific surface energy (SSE) was calculated using a unique AFM technique described in [61]. The contact angle was determined using a 2 μL drop of the electrolyte solution that was used for film deposition.

## 3. Results and Discussion

### 3.1. Surface Structure and Properties of the Substrates

The method of the surface preparation of the substrates can have a significant effect on adhesion between the substrate and the electrodeposited film. Particular attention should be paid to the adhesion between the substrate and the film in case of deposition on a polished surface. Adhesion tests by the lattice pattern method were carried out for all samples. The adhesion test results, in accordance with the Russian standard GOST 9.302-88 and American standard ASTM D 3359-09, showed that all samples had no peeling, removal, cracking, etc., with 0% area removed, and the edges of the cuts were completely smooth.

**Figure 2:**
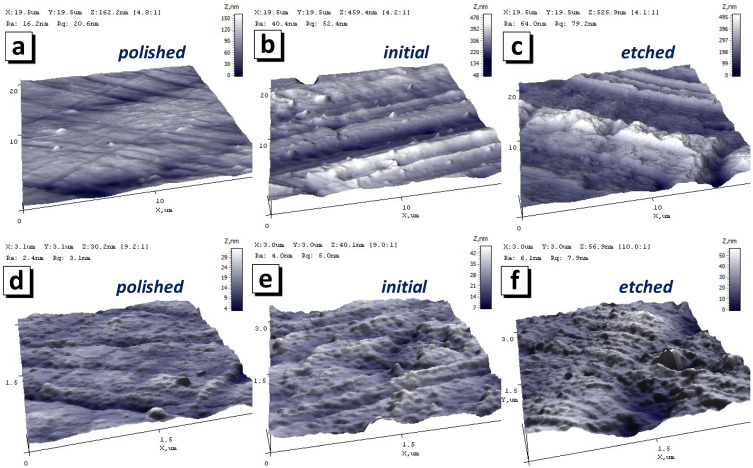
Three-dimensional AFM images of substrate surface topography after mechanical polishing (**a**,**d**), without special preparation or the initial substrate (**b**,**e**) and after mild chemical etching (**c**,**f**). The images sizes are 20 × 20 µm^2^ (**a**–**c**) and 3 × 3 µm^2^ (**d**–**f**).

Figure 2 shows AFM images of the three substrate types used at different magnifications (sizes of images are 20 × 20 µm^2^ (a–c) and 3 × 3 µm^2^ (d–f)), including for substrates polished with subsequent mild etching (a,d), after mild etching or initial substrate (b,e) and after intensive etching (c,f). The figure clearly shows that polishing followed by mild etching (Figure 2a,d) made the surface more uniform and smoother compared to the other samples. Table 2 shows the main substrate parameters used for deposition. It is important to distinguish between roughness levels. The surface roughness determined in a 3 × 3 µm^2^ area will differ from the roughness in a 20 × 20 µm^2^ area. For simplicity’s sake, the terms “nano-roughness” and “micro-roughness” will be used. The values of nano-roughness are always less than microroughness values [62]. The microroughness of the polished substrate was 22 nm, and the initial and etched micro-roughnesses were 100 and 83 nm, respectively. The nano-roughness of the polished substrate was 2.1 nm, and the initial and etched micro-roughnesses were 4.4 and 6.1 nm, respectively. The discrepancy between the values of micro- and nano-roughness is a consequence of the different nature of these parameters. In the case considered here, micro-roughness was determined by the depth of the grinding and polishing grooves and surface waviness; nano-roughness was determined by the configuration of nanosized grains on the surface of the samples.

An equally important parameter for the substrate electrodeposition is the ratio of the nominal area to the full area (R_N/F_) [60]. The ratio R_N/F_ allows for an estimation of the nature of the potential distribution on the surface during the reduction of metal ions on the substrate during electrodeposition. The lowest value of the R_N/F_ = 0.980 corresponds to the substrate that was subjected only to mild etching (initial). Conversely, after mechanical polishing, the substrate had the ratio closest to one (0.992). It was determined in an area of 20 × 20 µm^2^. Thus, the same value of the current strength set by the equipment during deposition for these substrates will lead to different values of the actual current density on the surface. The lowest current density will correlate to the initial substrate, and the highest will correlate to the polished substrate.

Another important substrate characteristic is wettability, or the contact angle value. The results of the contact angle measurement (Table 2) show that the polished substrate exhibited hydrophobic behavior concerning the electrolyte (contact angle = 83°), while other substrates (after mild and intensive etching) were more hydrophilic (contact angles were in range 68–69°). Specific surface energy (SSE) is closely related to wettability and is in agreement with it. As shown in Table 2, the SSE of the polished Cu substrate was 0.18 N/m, and after mild and intensive etching, the substrates had similar values of specific surface energy equal to 0.26 and 0.27 N/m, respectively.

### 3.2. Surface Structure and Properties of the NiFe Films

The deposition was carried out in a galvanostatic mode, and the voltage change during deposition can be observed in Figure 1. An increase in potential followed by a decrease to a constant value is a common phenomenon in the electrodeposition of binary and ternary alloys from complex electrolytes [43,63]. During the first stages of deposition (from 10s of seconds to 3–5 min), the potential increases due to an increase in the electrolyte resistance and a higher potential for the discharge of metal ions onto the substrate material. In addition, the growth of the potential is due to the difficult diffusion of heavy metal ions to the cathode. The decrease in the deposition potential is indicative of a change in the deposition kinetics in this region under the influence of a number of competing factors. After the extremum is reached, deposition occurs on a fully formed film, and the discharge potential decreases. This also affects the formation of complexes from simple salts in the electrolyte solution and the diffusion of ions to the cathode. As a result, the resistance of the electrolyte becomes low, the potential reaches a minimum and remains constant, and the deposition process is in equilibrium.

The thickness of the Ni-Fe films obtained within 1 min was about 300–400 nm. Films with a thickness of 1.0–1.2 µm were formed in 3 min, 3.5–3.7 µm in 10 min of growth and about 9 µm in 25 min.

Table 3 demonstrates results of XRD investigations of the NiFe films obtained on the different substrates during 25 min. The XRD study verified that all films were well-described by a cubic-face-centered structure, with a space group Fm-3m (No. 225). The parameter and unit cell volume of the NiFe film obtained on a polished substrate were 3.570 Å and 45.499 Å^3^, respectively. The parameters and volumes of the films obtained on the initial and etched substrates were equal to each other and amounted to 3.372 Å and 45.576 Å^3^. The smaller size of the unit cell of Polish-25 film may have been a consequence of the surface compression of nanosized crystallites. As the results show (see Table 3), the coherent scattering region (CSR) for the Polish-25 sample was 5.3 nm, while it was 6.1 and 6.0 nm for the Initial-25 and Etch-25 samples. For the same reason (surface compression of crystallites), an increase in internal microstrain was observed for the Polish-25 samples (0.39 %) as compared to the Initial-25 (0.28 %) and Etch-25 (0.29 %) samples.

**Figure 3:**
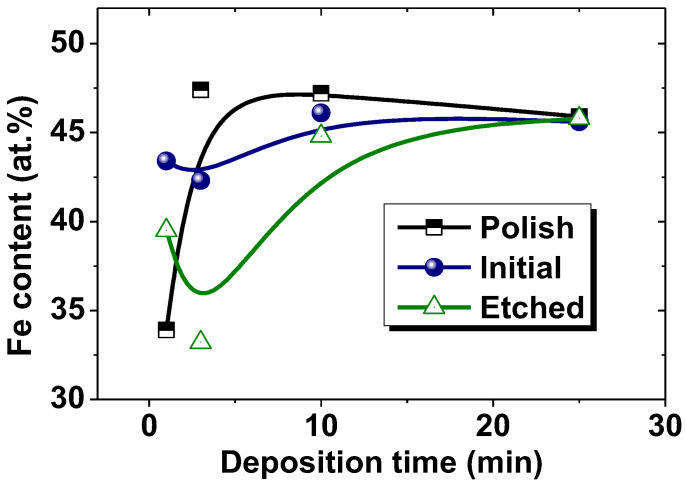
Iron concentration in NiFe films obtained on substrate after mechanical polishing (polish), without special preparation (initial) and after mild chemical etching (etch) depending on deposition time (1–25 min).

Figure 3 shows the change in the iron content in the NiFe films depending on the deposition time. It should be noted that the data show the composition on the film surface rather than the entire volume, as was achieved in EDX studies due to the low value of the accelerating voltage (5 kV) during investigation.

The NiFe films obtained within 1 min on polished, initial and etched substrates had an iron content of 33.9, 43.4 and 39.5 at.%, respectively (Figure 3). This can be fully explained by the influence of the structure and surface properties of the substrates. The polished substrate had the lowest roughness, a high ratio of the nominal to the full area and was highly hydrophobic (see Table 2). These characteristics, in combination, lead to the fact that the real surface area that was involved in the electrodeposition process was close to the nominal one (R_N/F_ = 0.992). The opposite was seen for the substrate characteristics after mild etching (initial). The roughness values were much higher, and the contact angles were smaller (hydrophilic properties) (Table 2). The etched substrate had similar values of wettability and roughness. The etched, and especially the initial substrate, had a larger real surface area than the nominal one (R_N/F_ = 0.980 for the initial substrate and R_N/F_ = 0.988 for the etched substrate). The result of this is that the current density during electrodeposition was higher on the polished surface than on the initial and etched substrates. It is known that an increase in the current density leads to a decrease in the iron content in the film, which was described in [43,45]. In addition, the high surface roughness of the initial substrate (with a micro-roughness equal to 100 nm and nano-roughness equal to 4.6 nm) and etched substrate (with a micro-roughness equal to 83 nm and nano-roughness equal to 6.0 nm) prevented the removal of hydrogen from the cathode surface, which is actively released during the electrodeposition process. A high concentration of H^+^ ions lead to a decrease in the pH level in the cathode region, which leads to an increase in iron content. It is known that a decrease in the pH level leads to the formation of iron-rich films [43,45].

**Figure 4:**
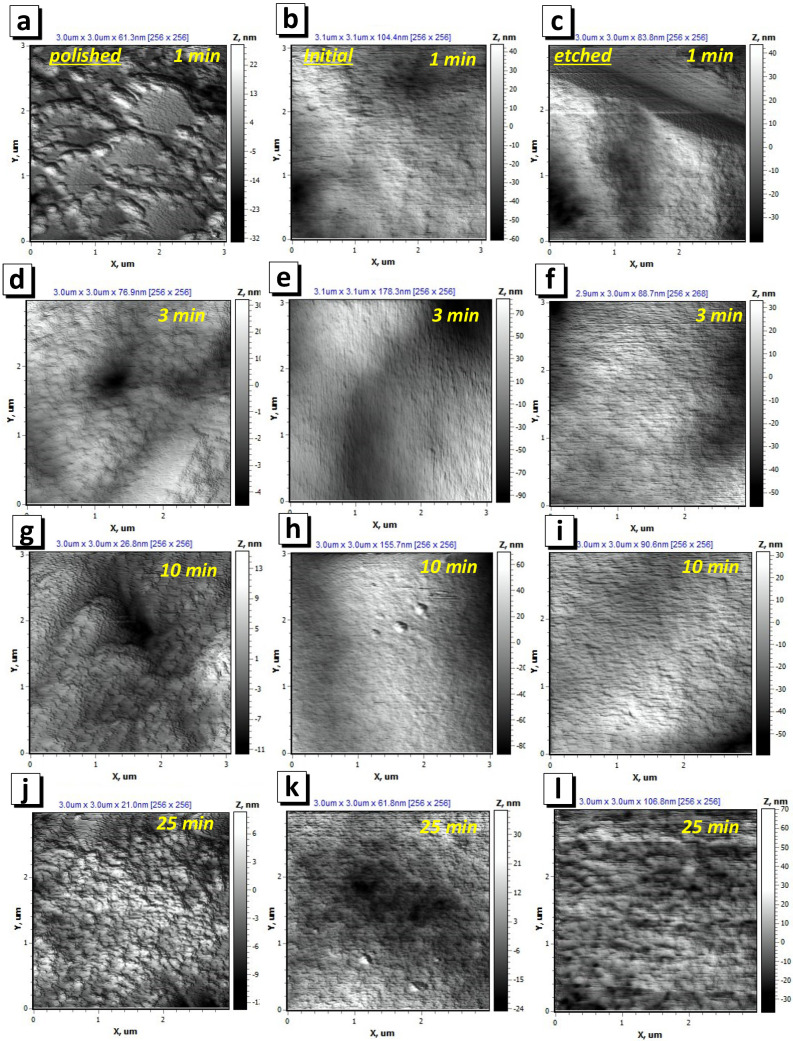
AFM images of topography of NiFe films on substrate after polishing (**a**,**d**,**g**,**j**), without special preparation or initial substrate (**b**,**e**,**h**,**k**) and after mild chemical etching (**c**,**f**,**i**,**l**). The deposition times were 1 min (**a**–**c**), 3 min (**d**–**f**), 10 min (**g**–**i**) and 25 min (**j**–**l**). The images sizes are 3 × 3 µm^2^.

**Figure 5:**
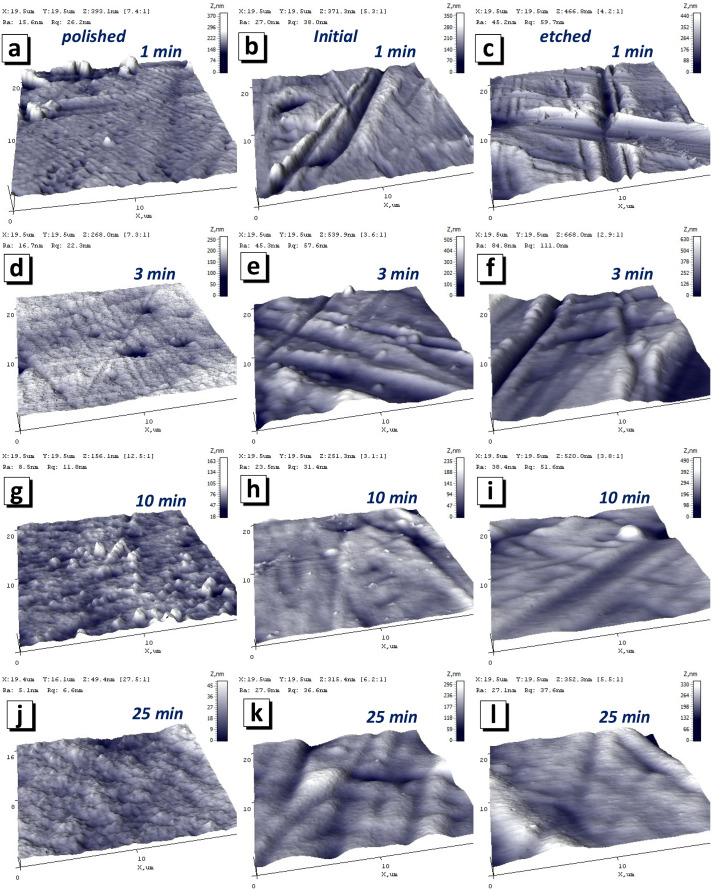
Three-dimensional AFM images of NiFe films on substrate after polishing (**a**,**d**,**g**,**j**), without special preparation or initial substrate (**b**,**e**,**h**,**k**) and after mild chemical etching (**c**,**f**,**i**,**l**). The deposition times were 1 min (**a**–**c**), 3 min (**d**–**f**), 10 min (**g**–**i**) and 25 min (**j**–**l**). The images sizes are 20 × 20 µm^2^.

**Figure 6:**
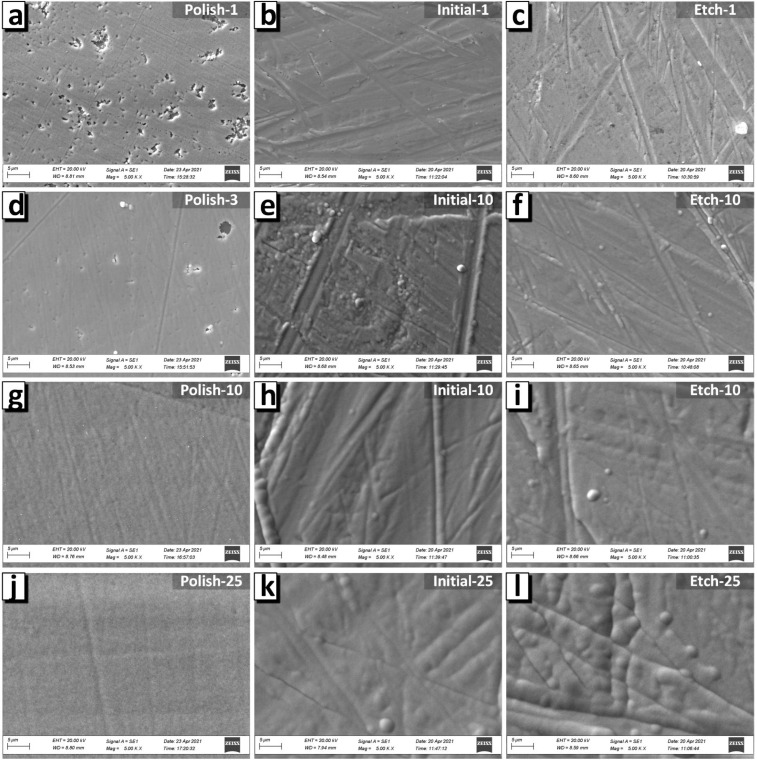
SEM images of the NiFe films on substrates with different roughnesses of substrate surface topography after polishing (**a**,**d,g,j**), without special preparation or initial substrate (**b**,**e,h,k**) and after chemical etching (**c**,**f,i,l**).

**Figure 7:**
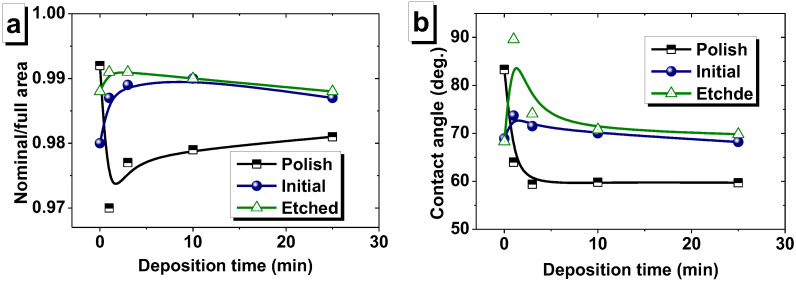
Ratio of nominal/full surface area (**a**) and contact angle (**b**) of NiFe films depending on deposition time.

As a result, a rough structure with clearly visible grains 100–200 nm in size was formed on the surface of the polished substrate (Figure 4a). It is likely that island growth appeared on the film, which is a consequence of the poor wettability of the substrate. The AFM image (Figure 5a) and SEM image (Figure 6a) have a lower magnification and show that a film with pores was formed on the polished substrate within 1 min of electrodeposition. This led to the ratio of the nominal to the full area (0.970) (Figure 7a). The grain structure of the surface naturally led to a decrease in the contact wetting angle from 83.3 to 54.0 deg. (Figure 7b). As a result, an increase in the iron content to 47.4 at.% (3 min) was observed with further film growth. With a further increase in the deposition time, the changes in wettability and R_N/F_ were not significant, and their effect was compensated for, so the composition of the deposited films remained constant with an iron content of about 46–47 at%.

The behavior of the films growing on the initial and etched substrates was opposite. The micro- and nanoscale bumps of the substrates were quickly filled with nanosized grains, and within 1 min of growth, smooth films with low roughness had already formed on the initial and etched surface. Thus, the ratio of the nominal to the full area of the initial-1 film was 0.987, and that of the etched-1 film was 0.991. Low roughness and good surface uniformity led to poor wettability. The contact angle was 73.7 and 89.6 deg. for the inital-1 and etched-1 films, respectively. Thus, with further growth of the films (3 min), iron-depleted layers were formed, which can be seen in the graph of the dependence of the iron content on the deposition time (Figure 3) for the initial and etched substrates. Films obtained on initial and etched substrates within 3 min, in contrast to those obtained in 1 min, had grains with a size of 2–4 μm on the surface (Figure 5e,f and Figure 6e,f). The presence of grains caused some decrease in wettability, which in turn led to an increase in the iron content to 46.1 and 44.8 at % for the films on the initial and etched substrates, respectively, as described above.

The SEM and AFM images (Figure 4, Figure 5 and Figure 6) show no significant changes in the film surface structure with a further increase in the deposition time and film thickness. Additionally, the values of the wetting angle and R_N/F_ did not change significantly with an increase in the deposition time from 3 to 25 min and the film thickness from 1.0–1.2 to 9 μm. Consequently, the composition of the films formed at a deposition time of more than 3 min remained unchanged.

## 4. Conclusions

NiFe films were electrolytically deposited onto Cu substrates, which were prepared for synthesis in various ways. As a result of different preparations, the substrates had different microstructure and surface properties. The study of the chemical composition gradient showed that the NiFe film obtained on a polished substrate had the lowest iron content (33.9 at.%), and the Fe concentration in the film increased to about 46–47 at.% for a film with a thickness of about 1 μm. Finally, the stoichiometry did not change during further growth on the polished substrate. NiFe films obtained on the initial substrate and the substrate after aggressive etching showed another behavior. At the first stages, the iron content in the film was 33.9 and 39.5 at.%, and then it decreased to its minimum. When the film thickness reached about 9 µm, the iron content in all samples was the same and equaled 46–47 at.%. Such anomalous behavior is explained by the complex and competing influence of the structure and properties of the surface. Firstly, the low ratio of the nominal area to the total area contributed to a decrease in the actual value of the current density. An increase in the current density led to a decrease in the iron content in the film. Secondly, the poor wettability of the substrates and the films already deposited by the electrolyte led to an increase in the current density and, consequently, to a decrease in the iron content. Finally, the high surface roughness prevented the removal of hydrogen from the cathode surface, which is actively released during the electrodeposition process. A high concentration of H^+^ ions led to a decrease in the pH level in the cathode region, which led to an increase in iron content. A decrease in the pH level led to the formation of iron-rich films.

## Figures and Tables

**Table 1:** Features of substrate preparation, electrodeposition parameters and sample descriptions.

Short Name	Substrate Material	Substrate Preparation	Electrolyte pH	Electrolyte Temperature, °C	Current Density, mA/cm^2^	Deposition Time, min
Polish-1	Cu	Mechanical polishing, mild HCl etching (5 s)	2.0	35	25	1
Polish-3						3
Polish-10						10
Polish-25						25
Initial-1	Cu	Mild HCl etching (5 s)	2.0	35	25	1
Initial-3						3
Initial-10						10
Initial-25						25
Etch-1	Cu	Intensive (NH_4_)_2_SO_8_ ammonium persulfate etching (60 s)	2.0	35	25	1
Etch-3						3
Etch-10						10
Etch-25						25

**Table 2:** Surface properties of Cu substrates with different preparation techniques.

Substrate	Substrate Micro-Roughness, nm	Substrate Nano-Roughness, nm	R_N/F_ *	Contact Angle, °	SSE **, N/m
Polished	22	2.1	0.992	83.3	0.18
Initial	100	4.6	0.980	68.9	0.26
Etched	83	6.0	0.988	68.3	0.27

* R_N/F_ is the ratio of the nominal to the full area, R_N/F_ = 1 for atomically rough (ideal) surface. ** SSE is a specific surface energy obtained by AFM.

**Table 3:** Crystal structure parameters of NiFe films deposited on substrates after different preparations during 25 min.

Sample	Unit Cell Parameter, a, Å	Cell Volume V, Å^3^	CSR *, nm	Microstrain, %
Polish-25	3.570	45.499	5.3	0.39
Initial-25	3.572	45.576	6.1	0.28
Etch-25	3.572	45.576	6.0	0.29

* CSR is the coherent scattering region.

## Data Availability

The data of the study are available upon reasonable request from the corresponding author.
